# Availability and readiness of the health facilities to provide HIV counseling and testing and prevention of mother-to-child transmission services in Burkina Faso: a trend analysis from 2012 to 2018

**DOI:** 10.1186/s12913-023-09757-1

**Published:** 2023-07-14

**Authors:** Lucresse Corine Fassinou, Hervé Hien, Jean Cyr Yombi, Nicolas Nagot, Fati Kirakoya-Samadoulougou

**Affiliations:** 1 Centre de Recherche en Epidémiologie, Biostatistiques Et Recherche Clinique, Ecole de Santé Publique, Université Libre de Bruxelles, Brussels, Belgium; 2 Institut National de Santé Publique, Ouagadougou, Burkina Faso; 3 Department of Internal Medicine, Infectious and Tropical Diseases, AIDS Reference Centre, Cliniques Universitaires Saint Luc, Université Catholique de Louvain, Brussels, Belgium; 4 Pathogenesis & Control of Chronic and Emerging Infections, University of Montpellier, INSERM, University of Antilles, Etablissement Français du Sang, Montpellier, France

**Keywords:** HIV counseling and testing, Prevention of mother-to-child transmission, Health facilities, Availability, Readiness, SARA, Burkina Faso

## Abstract

**Background:**

Provider-Initiated HIV Testing and Counseling (PITC) and Prevention of Mother-To-Child Transmission (PMTCT) are key services for achieving the goal of complete elimination of HIV. However, there is limited evidence on the ability of health facilities to provide these services in Burkina Faso. Therefore, we aimed to assess the trends and disparities in the availability and readiness of health facilities to provide PITC and PMTCT services in Burkina Faso between 2012 and 2018.

**Methods:**

We performed a secondary analysis of facility-level data from the World Health Organization’s Service Availability and Readiness Assessment (SARA) surveys conducted in 2012, 2014, 2016, and 2018 in Burkina Faso. The availability and readiness of health facilities were assessed using SARA’s manual, and linear regressions were used to examine trends.

**Results:**

Between 2012 and 2018, the mean proportion of health facilities providing PITC services increased, but not significantly, from 82.9% to 83.4% (*p* = 0.11), with the mean readiness index significantly decreasing from 71.5% to 65.4% (*p* < 0.001). This decrease concerned the staff and guidelines (73.8% to 50.5%; *p* < 0.001), equipment (79.0% to 77.4%; *p* < 0.001), and medicines and commodities (54.2% to 45.2%; *p* < 0.001) domains. Regarding the PMTCT services, the mean proportion of health facilities globally providing the service significantly decreased from 83.7% in 2012 to 67.7% (*p* = 0.030) in 2018, and the mean readiness significantly decreased from 53.2% in 2012 to 50.9% in 2018 (*p* = 0.004). This decreasing trend was related to the staff and training (80.3% to 57.6%; *p* < 0.001) and medicines and commodities (9.2% to 6.5%; *p* < 0.001) domains. The global significant negative trend of readiness was mainly observed at the primary level of healthcare (52.7% to 49.4%; *p* = 0.030). Four regions experienced a significant decrease in the readiness index of health facilities to provide PMTCT services: Cascades, Centre, Centre-Sud, and Sud-Ouest, while Haut-Bassins and Nord regions showed increasing trends.

**Conclusion:**

Availability and readiness of health facilities to provide PITC and PMTCT remain suboptimal in Burkina Faso. Actions to strengthen the skills of professionals and enhance the availability of medicines and commodities while focusing more on health regions with significant decreasing trends are urgently needed to improve the quality of services for HIV.

**Supplementary Information:**

The online version contains supplementary material available at 10.1186/s12913-023-09757-1.

## Background

In 2015, the Joint United Nations Programme on HIV/AIDS (UNAIDS) aimed for HIV testing, treatment and viral suppression rates to be 90%—90%—90% by 2020 [1]. Although these targets led to the global scale up of ART, progress has not been even across countries and populations as about 84% of all people living with HIV (PLWH) knew their status in 2020, and 87% of them were on treatment [1]. Achieving the new 95–95-95 targets set for 2030 thus require tailored interventions [2].

Sub-Saharan Africa (SSA) continues to bear the brunt of the HIV epidemic, with more than two-thirds (67%) of all PLWH residing in this region, despite only accounting for 12% of the global population [3, 4]. Recently, HIV care programs have greatly improved in SSA [5] with significant global investments in health services including the comprehensive packages for testing, prevention, treatment, and care services for at-risk populations, PLWH, their partners, families, and caregivers, as well as recommendations for health system strengthening [6].

Several of such programs were implemented in Burkina Faso, including the integration of maternal health services with HIV testing in 2002 [7], free distribution of effective ART across most health districts in 2010 [8, 9], the decentralization of medical care for PLWH in 2015 [10, 11], the adoption of the option B + in 2014 [12] and the free care policies for pregnant women and children under five in 2016 [13]. These different policies have effectively decreased the disease prevalence and vertical transmission in Burkina Faso from 1.1% and 31.6% in 2010 to 0.7% and 12% in 2020, respectively [14]. Despite this progress, more action is required to achieve the target of zero infections by 2030, and regular monitoring of healthcare facility readiness is essential to improve service availability.

The Service Availability and Readiness Assessment (SARA) is a tool jointly developed by the World Health Organization (WHO) and the UNAIDS to assess and monitor the availability and readiness of health facilities [15]. The SARA survey assesses the physical presence of services (service's availability) and the ability of health facilities to deliver services (health facilities' readiness) through the presence of “tracer items” necessary for providing services such as trained staff, guidelines, equipment, laboratory services, and medicine and commodities [16]. SARA surveys have been used in different settings [17–20]. A study conducted in Nepal to assess the availability and readiness of health facilities for sexually transmitted infections and HIV testing and counseling services using SARA tools found that HIV counseling and testing was available in less than 10% (5.9%) of the health facilities, with a readiness score of 68.9% [2]. Another multi-country study on the availability of integrated family planning in HIV care in SSA revealed that only 29% of the health facilities integrated this service globally, due to limited availability of guidelines and trained staff for offering the service [21]. These studies highlight that while some services are available, readiness scores are often low, emphasizing the importance of regular assessments to identify areas for improvement.

The elimination of HIV in Burkina Faso requires the provision of key services like the Provider-Initiated HIV Testing and Counseling (PITC) and Prevention of Mother-To-Child Transmission (PMTCT), especially among pregnant women and infants. The country has faced socio-political crises that may have impacted health services, making it necessary to assess the trend in service provision by health facilities over the years to understand the unmet care needs [22, 23]. SARA surveys, conducted in many SSA countries, provide excellent tools to assess general services and PITC and PMTCT services in particular [24]. However, the availability and readiness of health facilities to provide PITC and PMTCT services in Burkina Faso have not been assessed using these data. To achieve the goal of complete elimination of HIV, understanding the ability of health facilities to provide these services for targeted interventions and improvements is essential. This study aimed to evaluate trends in PITC and PMTCT services provided by health facilities in Burkina Faso from 2012 to 2018 and assess the disparities in patterns by characteristics of health facilities.

## Methods

### Study design and setting

We performed a secondary analysis of the data obtained from the SARA surveys. This analysis involved data collected from 2012 to 2018 in the different health facilities of Burkina Faso. The healthcare system of Burkina Faso is pyramidal and includes public, private, and traditional medicine and pharmacopeia subsystems. The public care structures are organized into three levels: the peripheral level constituted by health districts including the “Centre de Santé et de Promotion Sociale” (CSPS), medical centers, isolate dispensaries, delivery centers, and district hospitals which serve as referral centers of former health facilities; the intermediate level made of regional hospitals which are the reference structures for district hospitals; and the central level representing the highest level of referral care providing specialized services and comprising the national and teaching hospitals [25, 26]. The public care system is managed at three levels: the central level, organized around the office of the Minister of Health and the General Secretariat, which is responsible for policy development, resource mobilization, management control, and performance evaluation; the intermediate level, which consists of 13 regional health directorates responsible for coordination and support to the districts; and the peripheral level, which currently has 73 health districts whose core teams manage basic and primary health services [25]. Despite this structural organization, the use of health facilities and preventive and curative means in the country is still low (0.87 contacts per capita in 2014) [27].

Since 2012, the Ministry of Health has administered the SARA survey every two years to assess and monitor the availability and readiness of health facilities to provide quality health services [28]. This survey includes public and private healthcare facilities across the three levels of the healthcare system in Burkina Faso. Facilities were sampled using a single-stage stratified random sampling method designed to provide a representative national sample [20]. All health facilities at the central (national) and intermediate (regional) levels were grouped into stratum 1, those at primary level and scale 2 consisting of district hospitals and private clinics were grouped into stratum 2, and those at primary level and scale 1 which represent the peripheral health facilities were grouped into stratum 3. In contrast to strata 1 and 2 which included all health facilities, stratum 3 included peripheral facilities selected using a simple random sampling. The indicators were considered representative at the national and regional levels. The WHO SARA standard questionnaire was used for data collection. This questionnaire was administered to the heads of  health facilities or any other authorized person, by data collectors who were certified health workers. Experienced health workers in the healthcare practice with a high level of education were designed as team leaders. In addition to the interview, a direct observation method was also used to verify the availability and/or the functionality of the necessary items. Before starting each survey, the collection teams received comprehensive training in collection tools. The team leaders supported data collectors in the collection of data in the health facilities of strata 1 and 2. 

### Measurement of variables

#### Outcome variables

The dependent variables consisted of two groups: service's availability and health facility’s readiness. Service availability was defined in this study as health facilities offering PITC and PMTCT services. The health facility’s readiness was defined as the ability of the health facility to provide PITC and PMTCT services. According to the WHO manual, the capacity of facilities to provide PITC and PMTCT services was based on 6 and 18 items, respectively, as presented in Table 1. If the tracer was available, it was coded as 1, and otherwise, 0. To assess the readiness of health facilities, four domains were considered: staff and guidelines, equipment, diagnostics, and medicines and commodities. For the diagnostic domain, the tracer was coded 0 if none of the materials (HIV rapid kit or enzyme-linked immunoassay (ELISA) testing with ELISA washer, ELISA reader, incubator, and specific assay kit) were available and functioning and 1 if at least one was available and functioning. For the PMTCT service, in the medicines and commodities domain, maternal ARV prophylaxis was coded 1 if medicine was used in option A (zidovudine and nevirapine and lamivudine) or option B (at least one of the following combination: zidovudine + lamivudine + lopinavir; zidovudine + lamivudine + abacavir; zidovudine + lamivudine + efavirenz; zidovudine + lamivudine + nevirapine; tenofovir + lamivudine + efavirenz; tenofovir + emtricitabine + efavirenz) prophylaxis was available and valid. Otherwise, it was coded as 0.

**Table 1:** Tracer items for availability and readiness of health facilities to provide PITC and PMTCT services

Domains	PITC services	PMTCT services
**Service availability**	HIV counseling and testing	PMTCT services
		Counseling and testing for HIV + pregnant women
		Counseling and testing for infants born to HIV + women
		ARV prophylaxis to HIV + pregnant women
		ARV prophylaxis to infants born to HIV + women
		Infant and young children feeding counseling
		Nutritional counseling for HIV + women and their infants
		Family planning counseling to HIV + women
**Service readiness**		
Staff and guidelines	Guidelines on HIV counseling and testing	Guidelines for PMTCT
	Staff trained in HIV counseling and testing	Guidelines for infant and young children feeding counseling
		Staff trained in PMTCT
		Staff trained in infant and young children feeding
Equipment	Room with auditory/visual privacy for HIV	Room with auditory/visual privacy for HIV
Diagnostic	Availability of HIV rapid diagnostic kit or ELISA test with ELISA washer, ELISA reader, incubator, specific assay kit	Availability of HIV rapid Diagnostic kit or ELISA test with ELISA washer, ELISA reader, incubator, specific assay kit
		DBS filter paper with valid expiration date
Medicines and commodities	Condoms	Zidovudine syrup in stock with at least one valid
		Nevirapine syrup in stock with at least one valid
		Maternal ARV prophylaxis

#### Independent variables

The independent variables were constituted of:

- ❖ Level of health facility (primary, secondary, and tertiary);
- ❖ Type of health facility (public, private);
- ❖ Location of the health facilities (urban, rural);
- ❖ Health regions (Boucle du Mouhoun, Cascades, Centre, Centre-Est, Centre-Nord, Centre-Ouest, Centre-Sud, Est, Haut-Bassins, Nord, Plateau-Central, Sahel, and Sud-Ouest).

### Statistical analysis

The SARA manual by WHO was used as a reference to perform all analyses. Service availability was described as the percentage of healthcare facilities that offered PITC and PMTCT services. All necessary tracer items in the domains were evaluated. A mean availability score (availability index) for each service was determined based on the mean availability of tracer items. For the readiness of health facilities, the assessment was performed based on a score calculated for each domain as a mean percentage of availability of the tracer item within the domain. The global domain score was calculated using the following formula: n/N*100 (where n represented the number of items available in the healthcare facility and N represented the total number of items for that domain). Finally, a service readiness index was calculated and defined as the mean of all the domain scores. For example, let's consider PMTCT services which have four domains: ‘Staff and guidelines’, ‘Equipment’,’Diagnostic’, and ‘Medicines and commodities’. If the domain scores for each domain were a, b, c, and d, respectively, the readiness index was equal to: $\sum \mathbf{a},\mathbf{b},\mathbf{c},\mathbf{d}/4$ (where a, b, c, and d represented the scores and 4 was the number of domains). The calculation of service readiness include only health facilities where the services were available for the reason that a health facility could not be "ready" for a service that it does not provide.  

The crude trend of the availability of PITC and PMTCT was measured by the chi-square test for trend using the "ptrend" command in Stata. As for the readiness index, the trend was measured by adjusting for the health facilities' characteristics using a multivariate linear regression analysis. Multicollinearity was checked between the characteristics of health facilities after each regression analysis. Since the probability of selecting healthcare facilities differed depending on the level of the health facility in the healthcare system, a weighted analysis was performed. All analyses were performed using Stata Version 17.0 (Stata Corp, College Station, Texas, USA), and the significance threshold used was 5%. The overall availability and readiness for each survey year by region was mapped using QGIS version 3.18.2-Zürich software.

## Results

### Characteristics of health facilities

In total, 686 health facilities were surveyed in 2012, 766 in 2014, 677 in 2016, and 794 in 2018. More than 50% of these facilities in each survey were public, at the primary level in the healthcare system, and located in rural areas, as shown in Table 2.

**Table 2:** Characteristics of the health facilities surveyed, 2012–2018

Characteristics	2012 (*N* = 686)		2014 (*N* = 766)		2016 (*N* = 677)		2018 (*N* = 794)	
	**n**	**%**	**n**	**%**	**n**	**%**	**n**	**%**
**Type of health facility**								
Public	591	81.2	609	79.5	504	74.5	545	68.6
Private	95	13.8	157	20.5	173	25.5	249	31.4
**Level of health facility**								
Primary	586	85.4	659	86.0	567	83.8	653	82.2
Secondary	78	11.4	89	11.6	93	13.7	119	15.0
Tertiary	22	3.2	18	2.4	17	2.5	22	2.8
**Location of the health facilities**								
Urban	201	29.3	265	34.6	256	37.8	340	42.8
Rural	485	70.7	501	65.4	421	62.2	454	57.2
**Region**								
Boucle du Mouhoun	56	8.2	75	9.8	44	6.5	54	6.8
Cascades	39	5.7	30	3.9	36	5.3	44	5.5
Centre	94	13.7	143	18.7	142	21.0	145	18.3
Centre-Est	54	7.9	52	6.8	48	7.1	64	8.1
Centre-Nord	49	7.1	50	6.5	39	5.8	49	6.2
Centre-Ouest	54	7.9	69	9.0	55	8.1	59	7.4
Centre-Sud	43	6.3	34	4.4	38	5.6	41	5.2
Est	50	7.3	50	6.5	42	6.2	53	6.7
Haut-Bassins	66	9.6	84	11.0	76	11.2	89	11.2
Nord	53	7.7	65	8.5	46	6.8	55	6.9
Plateau	45	6.6	44	5.7	40	5.9	51	6.4
Sahel	38	5.5	33	4.3	37	5.5	44	5.5
Sud-Ouest	45	6.5	37	4.8	34	5.0	46	5.8

### Availability index of health facilities providing PITC and PMTCT services

**Fig. 1:**
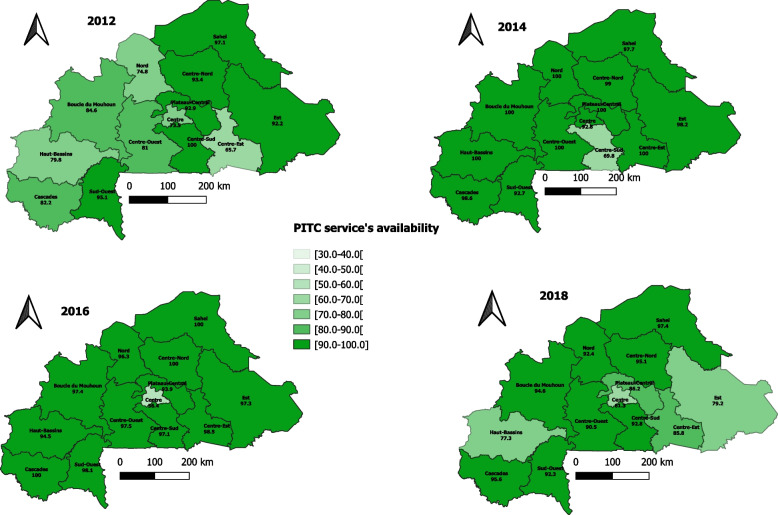
PITC service's availability by regions in Burkina Faso from 2012 to 2018

PITC service was available in 8 out of 10 health facilities (82.9%) in 2012, and this availability did not significantly change over the years, reaching 83.4% in 2018 (p-trend = 0.11) (Table 3). The trend significantly increased for public and private health facilities (p-trend < 0.001) from 87.7% in 2012 to 92.1% in 2018 for public settings and 51.3% to 65.3% for private health facilities. However, the proportion of health facilities providing this service remained somewhat stable, with values going from 83.5% in 2012 to 82.9% in 2018 for primary health facilities (p-trend = 0.043). Concerning the location of the health facilities, the proportion of health facilities located in urban areas significantly decreased from 70.2% in 2012 to 67.1% in 2018 (p-trend = 0.008), whereas for those located in rural areas, it significantly increased from 87.2% in 2012 to 95.2% in 2018 (p-trend < 0.001). Regarding the availability trend by region, a significant positive trend was noted for health facilities located in Boucle du Mouhoun (β =  + 0.018; p-trend < 0.001), Cascades (β =  + 0.011; p-trend = 0.019), Centre-Est (β =  + 0.034; p-trend < 0.001), Centre-Ouest (β =  + 0.017; p-trend < 0.001), Centre-Sud (β =  + 0.066; p-trend < 0.001), and Nord (β =  + 0.038; p-trend < 0.001) regions. In contrast, a negative trend was observed for the Centre (β = -0.028; p-trend < 0.001), Est (β = -0.021; p-trend < 0.001), and Plateau-Central (β = -0.012; p-trend = 0.024) regions. The availability according to the characteristics of health facilities and repartition of the proportions across the regions are presented in Table 4 and Fig. 1, respectively.

**Table 3:** Indicators of health facilities and their availability index to provide PITC and PMTCT services

Indicators	Years								
	**2012**		**2014**		**2016**		**2018**		**p-trend**^a^
	**n**	**%**	**n**	**%**	**n**	**%**	**n**	**%**	
**HIV counseling and testing**	578	82.9	714	93.8	597	90.8	660	83.4	0.11
**PMTCT services**									
PMTCT services offered	598	87.2	651	87.7	550	87.1	568	72.1	< 0.001
Counseling and testing for HIV + pregnant women	595	86.6	648	87.5	545	86.5	560	71.0	< 0.001
Counseling and testing for infants born to HIV + women	558	81.2	627	85.0	527	84.4	507	64.4	< 0.001
ARV prophylaxis to HIV + pregnant women	568	82.1	608	83.0	512	82.6	503	63.1	< 0.001
ARV prophylaxis to infants born to HIV + pregnant women	549	79.5	605	82.9	514	82.8	506	63.3	< 0.001
Infants and young child feeding counseling	577	84.0	638	86.3	530	85.0	548	69.3	< 0.001
Nutritional counseling for HIV + women and their infants	576	83.8	635	85.8	527	84.6	548	69.5	< 0.001
Family planning counseling to HIV + women	585	85.0	642	86.8	532	84.9	548	69.0	< 0.001
**PMTCT service availability index**	-	**83.7**	**-**	**85.6**	**-**	**84.7**	**-**	**67.7**	**0.030**

^a^Unadjusted p-trend

**Table 4:** Availability index of health facilities according to their characteristics

**Healthcare facilities’ characteristics**	**2012**	**2014**	**2016**	**2018**	**p-trend**^a^
**HIV Counseling and testing**					
**Type of health facility**					
Public	87.7	99.3	96.4	92.1	< 0.001
Private	51.3	63.5	62.0	65.3	< 0.001
**Level of health facility**					
Primary	83.5	93.8	90.9	82.9	0.043
Secondary	71.3	92.1	86.0	83.3	0.13
Tertiary	86.4	94.4	90.0	98.7	0.001
**Location of the health facilities**					
Urban	70.2	77.6	72.0	67.1	0.008
Rural	87.2	99.3	97.1	95.2	< 0.001
**PMTCT service’s availability index**					
**Type of health facility**					
Public	92.6	97.1	97.1	86.4	0.004
Private	24.6	22.4	23.3	28.7	0.78
**Level of health facility**					
Primary	84.9	86.8	86.0	72.0	< 0.001
Secondary	63.2	61.9	62.1	47.1	0.06
Tertiary	68.8	74.3	84.7	54.0	0.24
**Location of health facility**					
Urban	56.7	52.1	51.7	36.8	0.001
Rural	92.6	97.1	96.2	90.1	0.46

^a^Unadjusted p-trend

**Fig. 2:**
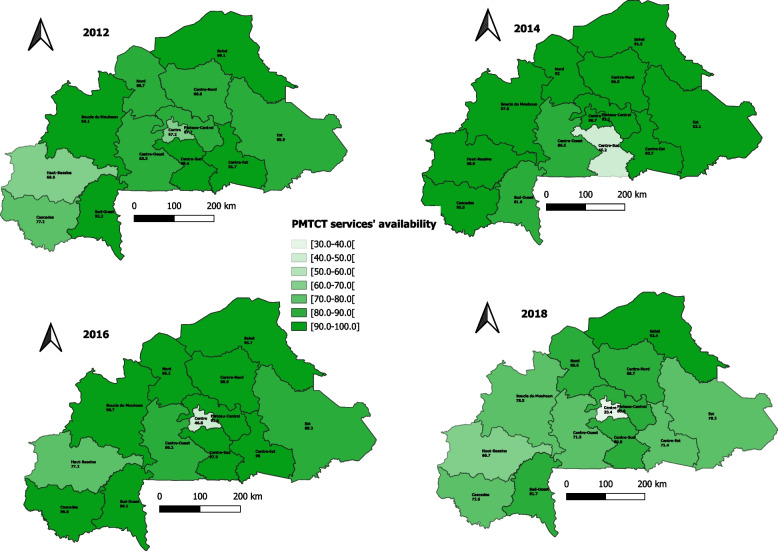
PMTCT services' availability index by regions in Burkina Faso from 2012 to 2018

The proportion of health facilities providing PMTCT services significantly decreased from 87.2% in 2012 to 72.1% in 2018 (*p* < 0.001). Globally, the mean score of PMTCT services' availability (taking into account all the indicators) also significantly decreased over the years, going from 83.7% in 2012 to 67.7% in 2018 (*p* = 0.030), as shown in Table 3. Even if this trend remained the same considering all the characteristics of health facilities, the decrease in mean score (availability index) was not statistically significant for the private facilities (*p* = 0.78), those belonging to the secondary and tertiary levels in the healthcare system (*p* = 0.06 and 0.24, respectively), and those located in rural areas (*p* = 0.46), as shown in Table 4. Concerning the region, only the Centre (β = -0.064; p-trend < 0.001) and Centre-Sud (β = -0.050; p-trend = 0.003) regions showed a significant decrease for the availability index; going from 67.2% in 2012 to 33.4% in 2018 for the Centre region and 99.4% in 2012 to 80.8% in 2018 for the Centre-Sud region (Fig. 2).

### Readiness index of health facilities to provide PITC services

The average readiness index for health facilities providing PITC service decreased significantly from 71.5% in 2012 to 65.4% in 2018 (p-trend < 0.001). Regarding the domains and tracers, a decrease was also observed for three of the four domains from 2012 to 2018: 73.8% to 50.5% for staff and guidelines domain, 79.0% to 77.4% for equipment domain, and 54.2% to 45.2% for medicines and commodities domain. For the diagnostic domain, a significant increase from 79.0% in 2012 to 88.5% in 2018 (p-trend < 0.001) was observed.

Concerning PMTCT services, the mean readiness index significantly decreased (p-trend = 0.004), from 53.2% in 2012 to 50.9% in 2018. The same decrease in trend was observed for each domain except for the equipment and diagnostic domains, for which a significant (p-trend < 0.001) increase was rather observed.

Tables 5 and 6 describe each domain and tracer besides the average readiness index of healthcare facilities providing PITC and PMTCT services.

**Table 5:** Domain score and readiness index of health facilities for PITC services

Domains and tracers	2012	2014	2016	2018	p-trend^a^
**Staff and guidelines**					
Guidelines on HIV counseling and testing	85.2	80.8	41.2	40.5	< 0.001
Staff trained in HIV counseling and testing	62.4	56.3	40.9	60.5	0.003
Mean domain score	73.8	68.5	41.0	50.5	< 0.001
**Equipment**					
Room with auditory and visual privacy for HIV	79.0	57.8	77.1	77.4	< 0.001
**Diagnostic**					
HIV rapid Diagnostic kit available or ELISA test with ELISA washer, ELISA reader, incubator, specific assay kit	79.0	90.7	83.3	88.5	< 0.001
**Medicines and commodities**					
Condoms	54.2	45.6	31.3	45.2	< 0.001
**Average readiness index**	71.5	65.7	58.2	65.4	< 0.001

^a^Trend was adjusted for type of health facility, level of health facility, location of the health facilities and health regions

**Table 6:** Domain score and readiness index of health facilities for PMTCT services

Domains and tracers	2012	2014	2016	2018	p-trend^a^
**Staff and guidelines**					
Guidelines for PMTCT	96.4	90.0	65.7	47.1	< 0.001
Guidelines for infants and young child feeding counseling	81.5	82.4	76.4	52.2	< 0.001
Staff trained in PMTCT	78.5	75.3	69.3	67.7	< 0.001
Staff trained in infants and young child feeding	65.0	67.0	88.3	63.4	0.004
**Mean domain score**	**80.3**	**78.7**	**74.9**	**57.6**	** < 0.001**
**Equipment**					
Room with auditory and visual privacy for HIV	79.2	58.0	76.7	80.8	< 0.001
**Diagnostic**					
HIV rapid Diagnostic kit available or ELISA test with ELISA washer, ELISA reader, incubator, specific assay kit	78.5	94.6	86.7	91.7	< 0.001
DBS filter paper with valid expiration date	9.3	17.3	16.3	23.0	< 0.001
**Mean domain score**	**43.9**	**57.1**	**52.2**	**58.6**	< 0.001
**Medicines and commodities**					
zidovudine syrup in stock with at least one valid	12.5	7.6	2.2	3.3	< 0.001
Nevirapine syrup in stock with at least one valid	1.1	15.0	5.8	7.0	0.002
Maternal ARV prophylaxis	14.2	12.1	6.3	9.1	< 0.001
**Domain mean score**	**9.2**	**11.6**	**4.7**	**6.5**	< 0.001
**Average readiness index**	**53.2**	**51.3**	**52.1**	**50.9**	**0.004**

^a^Trend was adjusted for type of health facility, level of health facility, location of the health facilities and health regions

### Readiness index for HIV counseling and testing and PMTCT services according to the characteristics of health facilities

**Fig. 3:**
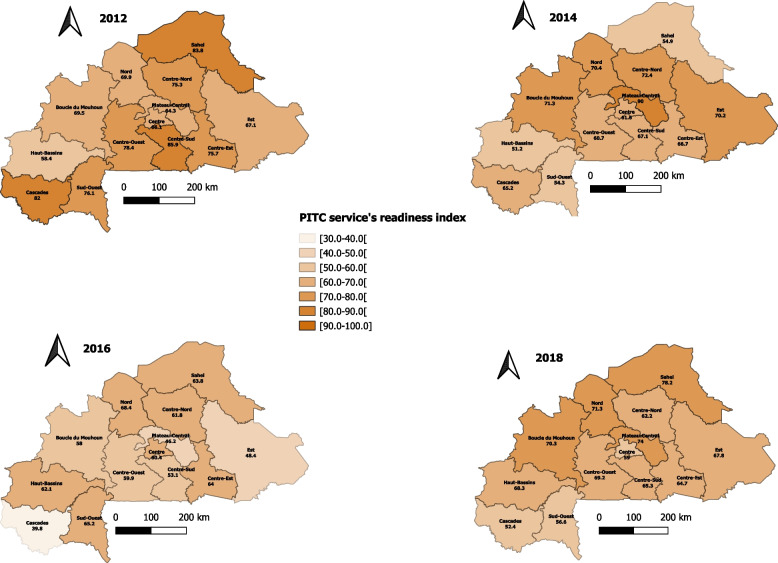
Readiness index of health facilities to provide PITC services from 2012 to 2018

Table 7 shows the change in the readiness of healthcare facilities to provide PITC and PMTCT services over the years, according to their characteristics. Considering the HIV counseling and testing services, the average readiness index significantly decreased from 72.4% in 2012 to 70.0% in 2018 (p-trend < 0.001) for public health facilities. A significant decrease was also noted at all levels of the healthcare system, from 71.2% to 65.7% (p-trend = 0.001) at the primary level, 76.9% to 65.9% (p-trend = 0.026) at the secondary level, and 75.0% to 57.2% (p-trend = 0.017) at tertiary level. A significant decrease was also observed in the readiness index based on location (urban: 69.5% in 2012 to 59.0 in 2018; p-trend = 0.011; rural: 72.0% in 2012 to 68.6% in 2018; p-trend = 0.004). Cascades (β = -8.142; *p* < 0.001), Centre-Nord (β = -4.736; p-trend = 0.002), Centre-Sud (β = -7.092; p-trend < 0.001), and Sud-Ouest (β = -3.809; p-trend = 0.025) regions presented significant decreases in the readiness of health facilities, with values moving from 82.0% (2012) to 52.4% (2018), 75.3% (2012) to 62.2% (2018), 85.9% (2012) to 65.3% (2018), and 76.1% (2012) to 56.6% (2018), respectively. By contrast, the Haut-Bassins region (β =  + 4.693; p-trend = 0.002) presented an increase in readiness index from 58.4% (2012) to 68.3% (2018), as shown in Fig. 3.

**Table 7:** Readiness index of health facilities to provide HIV counseling and testing and PMTCT services according to their characteristics

Domains and tracers	2012	2014	2016	2018	p-trend
**HIV counseling and testing**					
**Type of health facility**^a^					
Public	72.4	67.7	59.2	70.0	0.001
Private	60.9	48.0	49.9	51.7	0.07
**Level of health facility**^b^					
Primary	71.2	65.3	57.7	65.7	0.001
Secondary	76.9	72.3	66.0	65.3	0.026
Tertiary	75.0	68.4	62.8	57.2	0.017
**Location of the health facilities**^c^					
Urban	69.5	60.9	56.5	59.0	0.011
Rural	72.0	66.9	58.6	68.6	0.004
**PMTCT services**					
**Type of health facility**^a^					
Public	53.0	51.4	52.3	51.8	0.13
Private	55.6	49.3	49.2	46.0	0.021
**Level of health facility**^b^					
Primary	52.7	50.6	51.7	49.4	0.030
Secondary	61.8	66.4	60.7	57.8	0.58
Tertiary	67.4	72.5	55.0	72.9	0.72
**Location of the health facilities**^c^					
Urban	59.8	56.5	54.0	54.6	0.053
Rural	51.8	50.3	51.8	49.6	0.16

^a^Trend was adjusted for the level of health facility, location of the health facilities and health regions

^b^trend was adjusted for type of health facility, location of the health facilities and health regions

^c^trend was adjusted for type of health facility, level of the health facilities and health regions

**Fig. 4:**
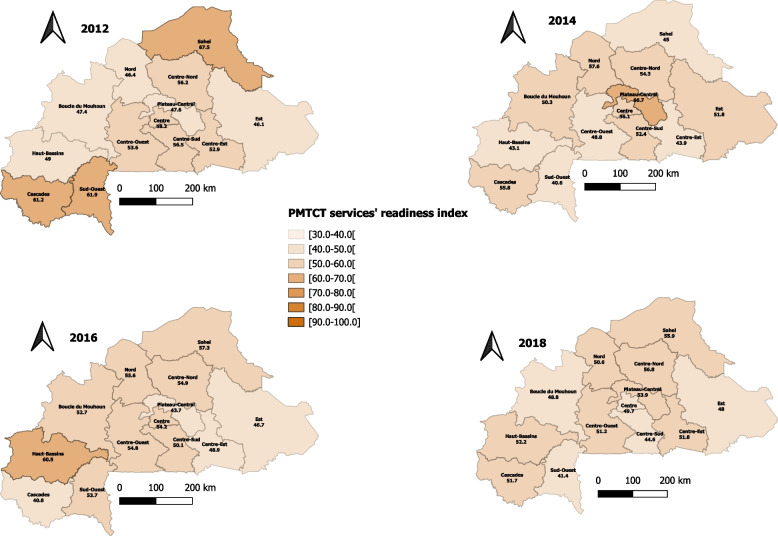
Readiness index of health facilities to provide PMTCT services from 2012 to 2018

Considering PMTCT services, the readiness index significantly decreased from 55.6% in 2012 to 46.0% in 2018 (p-trend = 0.021) for private health facilities. The readiness of health facilities belonging to the primary level significantly decreased, from 52.7% in 2012 to 49.4% in 2018 (p-trend = 0.030). Regarding the location, health facilities in both urban and rural areas presented a non-significant decrease in readiness index (p-trend = 0.053 and 0.016, respectively). Four regions experienced significant decrease in the readiness index of health facilities to provide PMTCT services: Cascades (β = -0.043; p-trend < 0.001), Centre (β = -0.029; p-trend = 0.001), Centre-Sud (β = -0.038; p-trend < 0.001), and Sud-Ouest (β = -0.036; p-trend = 0.007), with values changing from 61.2% (2012) to 51.7% (2018), 58.2% (2012) to 49.7% (2018), 56.5% (2012) to 44.6% (2018), and 61.9% (2018) to 41.4% (2018), respectively. The Haut-Bassins (β =  + 0.042; p-trend < 0.001) and Nord (β =  + 0.022; p-trend = 0.042) regions showed increasing trends, with values going from 49.0% (2012) to 52.2% (2018) and 46.4% (2012) to 50.6% (2018), respectively. Figure 4 shows the repartition by year and region of the readiness index of healthcare facilities in detail.

## Discussion

Like many other countries, Burkina Faso aims to accelerate the response to HIV and achieve the ambitious target of ending the epidemic by 2030 [11]. Thus, even though a significant improvement has been observed in recent years concerning the testing and treatment of PLWH [11], understanding the capacity of health facilities to provide HIV services is imperative to achieve the targets effectively since service readiness is a prerequisite to the delivery of quality healthcare [2]. Our study provided a comprehensive understanding of the changes in the availability and readiness of healthcare facilities to deliver PITC and PMTCT services in Burkina Faso from 2012 to 2018.

This study found no significant variation over the years in the availability of PITC service for the general population, unlike the PMTCT services, for which the availability significantly decreased over the years. The evaluation of the global availability of PMTCT services through an availability index also showed a significant decreasing trend during the same period. All the tracers of availability, such as the PMTCT service offered, counseling and testing for HIV + women, counseling and testing for infants born to HIV + women, ARV prophylaxis for HIV + pregnant women and infants born to these women, nutritional counseling for HIV + women and their infants, and family planning counseling for HIV + women, showed a significant decrease from a relatively high-level of availability in 2012 to a moderate level in 2018. This global negative trend remained the same for all types of health facilities except for private facilities, for which a slight non-significant increase was observed. The probable explanation for this could be that the political situation of the country has been worsening since 2015 [29]. Regarding PITC service, despite the global non-significant variation of this service’s availability in health facilities, some intra-variations according to the characteristics of health facilities were found in our study. A significant positive trend was observed for the two types of health facilities existing in the country (public and private), for facilities belonging to the tertiary level in the healthcare system, and for those in rural settings. Conversely, a significant decrease was noted for the facilities located in urban areas (70.2% in 2012 to 67.1% in 2018). One explanation for this surprising trend could be the availability of health professionals to provide counseling services. Healthcare providers could be extremely busy in urban settings, often due to the number of visits and the multiplicity of pathologies to be managed simultaneously; in the context of a shortage of healthcare human resources in Burkina Faso [30].

Among the facilities where PITC and PMTCT services were available, a significant decrease reflecting unsatisfactory readiness of health facilities to provide these services was observed, which could hinder strong progress that is continuously made to achieve the global target of elimination of HIV in the country. This decrease could be explained for PITC by the fact that the health facilities surveyed did not constitute all the screening providers; anonymous and free screening centers, as well as private laboratories, are major providers of HIV testing [31].

Specifically, considering PITC service, all domains encountered a significant decrease from 2012 to 2018. However, the decrease was most pronounced in the staff and guidelines domain, with a drastic reduction of almost half of the proportion of healthcare facilities with guidelines on HIV counseling and testing observed between 2012 and 2018. In addition, the availability of condoms decreased significantly over the years. In an African setting where male condoms constitute the cornerstone of HIV prevention campaigns [32], their optimal availability should be continuously maintained. This ensures that efforts in the fight against HIV are not undermined by the gap in availability. In 2017, Oyekale et al. reported the same result of non-readily available male condoms in primary health facilities in Nigeria [33]. The same observation of a decrease in the availability of guidelines was noted for the PMTCT services, for which guidelines were present in only 47.1% of health facilities in 2018 compared to 96.4% in 2012. This lack of guidelines could lead to suboptimal delivery of health services since no standard operational procedures existed to be followed by practitioners. External regular supervision could be a solution to address this gap, as it has been presented as an effective tool for improving the availability of guidelines [2]. In addition, a significant decrease in trained staff in both PITC and PMTCT services was noted; which was an important gap in the process of elimination of the epidemic. The availability of trained staff is key in ensuring that all requisite HIV services are delivered [34]. Welty et al. showed that trained staff significantly increased voluntary HIV screening and counseling [35]. Acharya et al. in 2020 had the same observations as ours concerning the lack of guidelines and trained staff as gaps in the readiness of health facilities to provide HIV counseling and testing in Nepal [2]. Regarding PMTCT services, out of the four domains, the staff and guidelines domain and medicines and commodities domain showed a significant decrease in the readiness index. In fact, unlike the significant increase in the availability of valid stock of nevirapine syrup, the proportion of health facilities with valid zidovudine syrup stock has significantly decreased, raising a considerable challenge in the prophylactic management of HIV in children born to HIV-positive women for the prevention of vertical transmission of the disease. Looking more specifically into the medicine and commodities domain (Appendix), the average availability of medicine for HIV prevention has significantly decreased from 2012 to 2018, with only 2.0% of ARV (children and adults) available in 2018 at the primary level, whereas more than half of the population received care from primary health facilities [36]. This significant decrease in the medicines availability may be explained by the low inventory management capacity of healthcare workers [37]. In addition, the decrease in the availability of ARV obtained at the primary level was more important in 2016, which could be explained by the socio-political and judicial crisis in 2016 of Burkina Faso’s central medical supplier, Centrale d’achat des medicaments essentiels génériques et des consommables médicaux (CAMEG), which caused an important shortage of essential medicines, especially in rural areas [38]. The low readiness of health facilities to provide medicines could be one explanation for the high prevalence (12%) of vertical HIV transmission [14].

Regarding the characteristics, a significant decrease was reported over the years for PITC and PMTCT services in primary health facilities. Our finding was in line with that of Crowley et al. in South Africa, who reported an inadequate capacity of primary healthcare centers to provide integrated HIV care and treatment services [39]. Since more than half of the population in Burkina Faso lives in rural areas and, therefore, has access to primary healthcare facilities [36], efforts that have already been made should be strengthened so that access to HIV care could be improved.

Over the years, the availability and readiness of healthcare facilities to provide both PITC and PMTCT services widely varied across regions in Burkina Faso. Differences in the tendency were observed between regions, which could be explained by the fact that technical and financial partners left many regions due to the security crisis in the country, which could have hampered the implementation of many activities and, thus, contributed to the decline in many assessment indicators. Indeed, Burkina Faso has been afflicted by an increased number of violent events related to terrorism (terrorism attacks) since 2015, with northern and eastern border areas being mostly but not exclusively, affected [40]. These attacks affect both the demand and provision of healthcare services. For example, the number of non-functioning health facilities has been increasing in the country owing to the abandonment of health facilities in the affected areas by health staff or the reduction of their activities in these areas [40, 41]. In maternal care, it has been shown that due to the insecurity in the country, the number of antenatal visits has been reduced by 1.8% for every additional attack in a commune [40]. The fewer people ask for the service, the less functional is the service, which explains why some regions are facing a decrease in indicators for PITC and PMTCT services.

In addition, based on our practical knowledge, the resources allocated to health facilities considerably decreased after 2014, and since the adoption of free care in 2016 to women and children under five years [42], these resources are more concentrated on the implementation of this policy. Financial limitations could be a reason for the discrepancy observed in our study.

Our results revealed that the Centre region faces a problem related to the low availability and readiness of health facilities to provide PMTCT services. This result which seems to be controversial since the region shelters the political capital of the country and is thus supposed to have well-equipped health facilities could be explained by the fact the region is mostly (68%) composed of private facilities [43]; while our results have shown a significant decrease in the readiness index for the private facilities. This suggests that full integration of private facilities, especially in the Centre region, for the provision of PMTCT services, must urgently be undertaken to improve the indicators. Another result found in our study is that despite the relative increase in the availability of the two services (PITC and PMTCT) in the Cascades region, the readiness index significantly decreased over the years. These results suggest that more investigations should be conducted in the different regions to deeply understand the difficulties encountered and improve the indicators so that quality services can be offered to the population.

### Strengths of the study

Our study is the first to focus on the trend of the availability and readiness of health facilities to provide PITC and PMTCT services in Burkina Faso. Four surveys were considered in the analysis to explore the trend over six years (2012–2018), as the SARA survey was conducted every two years. The surveys included in our study were representative of all 13 health regions of the country, which allowed us to see, in more detail, the trend across the regions and identify subnational disparities in the availability and readiness to provide HIV services. Moreover, since Burkina Faso’s health system is almost the same as that of most SSA countries, our study could help policymakers make decisions based on the results obtained.

### Limitations of the study

The data were collected by trained data collectors and were based on observations made in health facilities. However, some indicators such as “availability of HIV counseling and testing” or “availability of PMTCT services” were based on the declaration of the respondents and could have brought the response bias if the respondent was not qualified or lacked some information about the facility’s functioning. Another limitation of the study is its cross-sectional nature. It does not provide information on the duration of medicines disruptions, which is an important parameter when assessing service's availability and readiness, because it can help better understand the frequency, duration, and impacts of service interruptions in health facilities. Thus, it should be considered while developing interventions to improve availability and readiness of health facilities to provide services.

## Conclusion

This study reveals that the availability and readiness of health facilities to provide PITC and PMTCT remains suboptimal in Burkina Faso. Globally, a negative trend was observed in the availability of PMTCT services and the readiness of facilities to provide both PITC and PMTC services. These trends were related to the staff and guidelines, and medicines, suggesting that health policymakers should concentrate their efforts on these domains, especially in primary healthcare, to strengthen the skills of healthcare professionals so that quality services are offered. Consequently, it is key to improving the uptake of the services by the population. We also recommend that regions, where a significant decrease was observed in the availability and readiness of health facilities for each of the two services, are better monitored and supported. Thereby, all regions could have an equal improvement in service readiness across the country and progress toward the achievement of the 95–95-95 target set for 2030.

## Supplementary Information

**Additional file 1: Table S1.** PMTCT Medicine and commodities domain availability according to the characteristics of health facilities.

## Data Availability

The data that support the findings of this study are available from the “Direction Générale des Etudes et des Statistiques Sectorielles” of the Ministry of Health (MOH) of Burkina Faso, but restrictions apply to the availability of these data, which were used under license for the current study, and so are not publicly available. Data are, however, available from the authors upon reasonable request and with permission of the “Direction Générale des Etudes et des Statistiques Sectorielles” of the Ministry of Health (MOH) of Burkina Faso.

## Abbreviations

CAMEG: Centrale d’achat des medicaments essentiels génériques et des consommables médicaux
ARV: Antiretroviral drug
PITC: Provider-Initiated HIV Testing and Counseling
PLWH: Persons Living With HIV
PMTCT: Prevention of Mother-To-Child Transmission
SARA: Service Availability and Readiness Assessment
SSA: Sub-Saharan Africa
UNAIDS: Joint United Nations Programme on HIV/AIDS
USAID: United State Agency for International Development
WHO: World Health Organization
