# Supplementary material for: Availability and readiness of the health facilities to provide HIV counseling and testing and prevention of mother-to-child transmission services in Burkina Faso: a trend analysis from 2012 to 2018
Source: BMC Health Serv Res. 2023 Jul 14;23:757. doi: 10.1186/s12913-023-09757-1 (PMC10347843; doi:10.1186/s12913-023-09757-1)
Supplement: Supplementary file 1 — Additional file 1: Table S1. PMTCT Medicine and commodities domain availability according to the characteristics of health facilities. [file 12913_2023_9757_MOESM1_ESM.docx]

**Table S_1_.** PMTCT Medicine and commodities domain availability according to the characteristics of health facilities.

| Domains and tracers | 2012 | 2014 | 2016 | 2018 | p-trend* |
| --- | --- | --- | --- | --- | --- |
| **PMTCT medicines and commodities** |  |  |  |  |  |
| **Type of health facility** |  |  |  |  |  |
| Public | 9.0 | 11.6 | 4.4 | 6.6 | <0.001 |
| Private | 13.5 | 10.6 | 10.9 | 6.0 | 0.21 |
| **Level of health facility** |  |  |  |  |  |
| Primary | 8.4 | 10.1 | 2.9 | 2.0 | <0.001 |
| Secondary | 21.7 | 39.6 | 35.7 | 29.7 | 0.044 |
| Tertiary | 49.0 | 73.8 | 53.1 | 65.0 | 0.52 |
| **Location of the health facilities** |  |  |  |  |  |
| Urban | 12.0 | 20.5 | 15.7 | 21.2 | 0.21 |
| Rural | 8.7 | 9.9 | 2.6 | 1.7 | <0.001 |

*p-trend adjusted for region, type of health facility, location of health facility, and level in the healthcare system
